# Catching the Missing Million: Experiences in Enhancing TB & DR-TB Detection by Providing Upfront Xpert MTB/RIF Testing for People Living with HIV in India

**DOI:** 10.1371/journal.pone.0116721

**Published:** 2015-02-06

**Authors:** Neeraj Raizada, Kuldeep Singh Sachdeva, Achuthan Sreenivas, Shubhangi Kulsange, Radhey Shyam Gupta, Rahul Thakur, Puneet Dewan, Catharina Boehme, Chinnambedu Nainarappan Paramsivan

**Affiliations:** 1 Foundation for Innovative New Diagnostics, New Delhi, India; 2 Central TB Division, Government of India, New Delhi, India; 3 World Health Organization, India Country Office, New Delhi, India; 4 Foundation for Innovative New Diagnostics, Geneva, Switzerland; Fundació Institut d’Investigació en Ciències de la Salut Germans Trias i Pujol. Universitat Autònoma de Barcelona. CIBERES, SPAIN

## Abstract

**Background:**

A critical challenge in providing TB care to People Living with HIV (PLHIV) is establishing an accurate bacteriological diagnosis. Xpert MTB/RIF, a highly sensitive and specific rapid tool, offers a promising solution in addressing these challenges. This study presents results from PLHIV taking part in a large demonstration study across India wherein upfront Xpert MTB/RIF testing was offered to all presumptive PTB cases in public health facilities.

**Method:**

The study covered a population of 8.8 million across 18 sub-district level tuberculosis units (TU), with one Xpert MTB/RIF platform established at each TU. All HIV-infected patients suspected of TB (both TB and Drug Resistant TB (DR-TB)) accessing public health facilities in study area were prospectively enrolled and provided upfront Xpert MTB/RIF testing.

**Result:**

2,787 HIV-infected presumptive pulmonary TB cases were enrolled and 867 (31.1%, 95% Confidence Interval (CI) 29.4‒32.8) HIV-infected TB cases were diagnosed under the study. Overall 27.6% (CI 25.9–29.3) of HIV-infected presumptive PTB cases were positive by Xpert MTB/RIF, compared with 12.9% (CI 11.6–14.1) who had positive sputum smears. Upfront Xpert MTB/RIF testing of presumptive PTB and DR-TB cases resulted in diagnosis of 73 (9.5%, CI 7.6‒11.8) and 16 (11.2%, CI 6.7‒17.1) rifampicin resistance cases, respectively. Positive predictive value (PPV) for rifampicin resistance detection was high 97.7% (CI 89.3‒99.8), with no significant difference with or without prior history of TB treatment.

**Conclusion:**

The study results strongly demonstrate limitations of using smear microscopy for TB diagnosis in PLHIV, leading to low TB and DR-TB detection which can potentially lead to either delayed or sub-optimal TB treatment. Our findings demonstrate the usefulness and feasibility of addressing this diagnostic gap with upfront of Xpert MTB/RIF testing, leading to overall strengthening of care and support package for PLHIV.

## Background

Globally, an estimated 35.3 (32.2–38.8) million people are living with HIV [1]. TB is the leading cause of death among people living with HIV, accounting for 25% of HIV-related deaths. Persons co-infected with TB and HIV are more likely to develop active TB disease than persons without HIV infection [2]. In 2012, of the 8.7 million people who developed TB worldwide, 1.1 million (13%) were HIV infected. Further, of the 1.3 million TB deaths, 320, 000 were among people living with HIV [3]. India is the highest TB burden country accounting for 25% of global incidence and 4.85% of the incident TB cases are estimated to be among HIV infected [3]. Approximately 130,000 HIV associated TB cases emerge in India annually, out of which 42,000 co-infected patients die each year [4].

In order to address the issue of high mortality due TB/HIV co-infection in India, TB/HIV collaborative activities are being undertaken in India employing a differential strategy considering the heterogeneity of TB/HIV epidemic across the country. These collaborative activities include, intensified TB case findings at facilities providing care and support for HIV infection and risk-based referral of TB patients for voluntary HIV counselling and testing and referral of HIV-infected TB patients to facilities providing ART and TB treatment are undertaken [5].

However, a critical challenge in providing TB care to People Living with HIV (PLHIV) is establishing an accurate bacteriological diagnosis of TB. As HIV related immune-suppression increases, the clinical pattern of TB disease changes, with increasing numbers of smear-negative and extra pulmonary cases [6]. Sputum smears tend to be negative, as tubercle bacilli do not appear in sputum because of the paucity of pulmonary inflammation at early onset of disease and decreased cavitation. Further, though TB is the most common opportunistic infection among PLHIV, clinical decision-making is complicated because HIV infection broadens the scope of differential diagnosis of smear-negative pulmonary TB to include diseases such as *Pneumocystis carinii* pneumonia (PCP), pulmonary Kaposi's sarcoma, and Gram-negative bacteremia [7]. Furthermore, up to one third of HIV-TB co-infected cases might have completely normal chest radiographs due to less cavitation leading to increased chances of under diagnosis or missed diagnosis of TB in such cases [8]. Culture of sputum for *M*. *tuberculosis* though considered as the gold standard, is difficult to use and in resource-limited settings challenging to implement [9]. Culture result provided after 2–8 weeks are not available to guide immediate treatment decision-making needs [10]. Also, the role of Line Probe Assay is also limited in cases of smear negative TB patients for the diagnosis of DR-TB [11].

Xpert MTB/RIF, a high sensitive and specific tool with a quick turn-around time, offers a promising solution to address these challenges in the diagnosis of TB in HIV-infected patients. In 2013, WHO has released revised policy guidelines on the use of Xpert MTB/RIF in adults and children. These guidelines recommend that Xpert MTB/RIF should be used rather than conventional microscopy, culture and DST as the initial diagnostic test in adults suspected of having MDR-TB or HIV-associated TB (strong recommendation, high-quality evidence). They also recommend that Xpert MTB/RIF should be used rather than conventional microscopy, culture and DST as the initial diagnostic test in children suspected of having MDR-TB or HIV-associated TB (strong recommendation, very low-quality evidence) [12]. We have conducted a demonstration study to collect evidences on the feasibility and impact of implementation of upfront testing on Xpert MTB/RIF for the diagnosis of TB and rifampicin resistant TB cases, with specific focus on PLHIV, the related diagnostic and treatment delays, and treatment outcomes. In this article, we share evidence and key findings from our experiences of offering Xpert MTB/RIF for diagnosis of TB and drug resistant TB in HIV-infected TB cases, in programmatic settings at peripheral levels in India.

## Methods

### Setting

**Fig 1:**
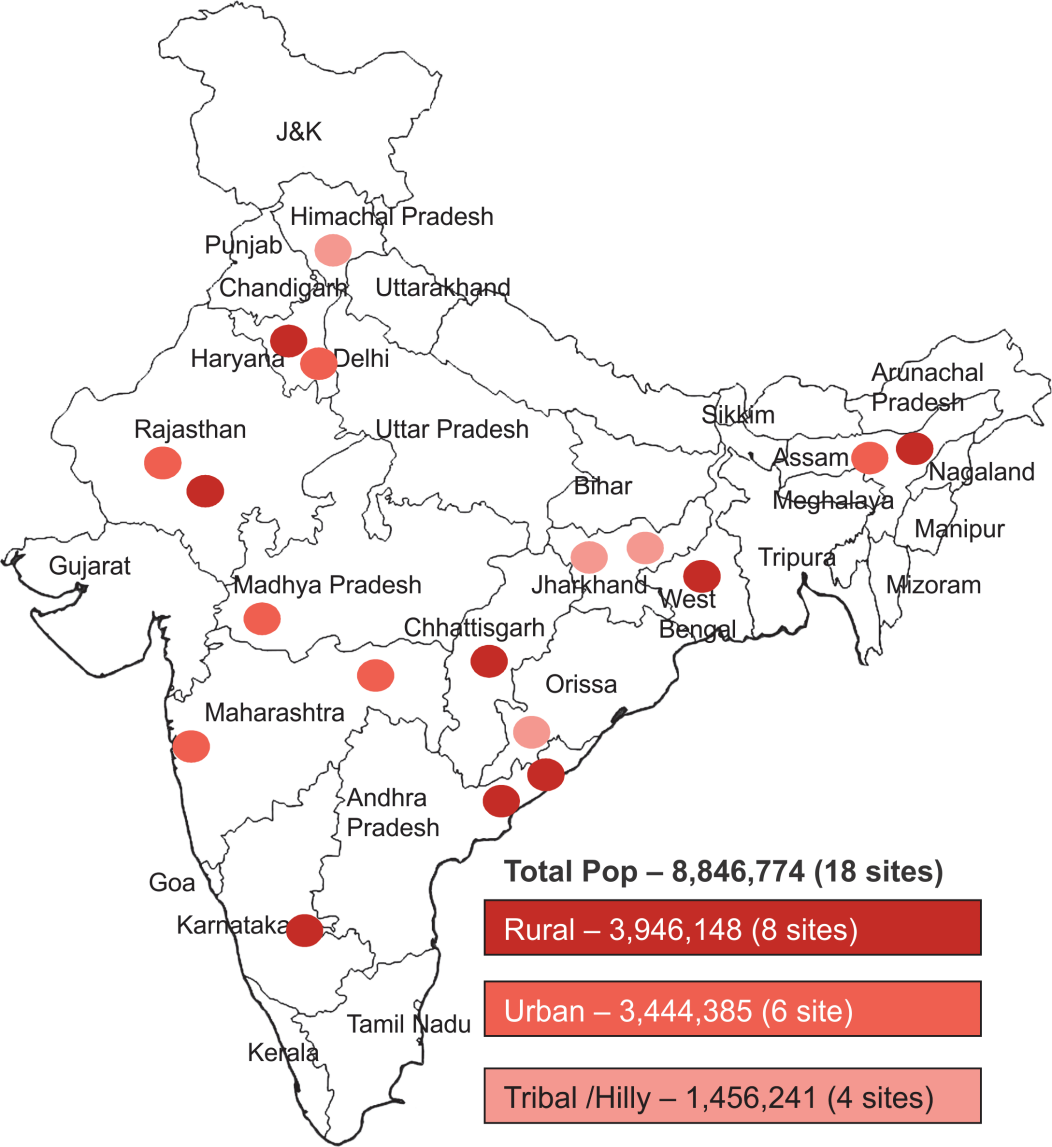
Geographical location of study treatment units and the demographic classification assigned to each project treatment unit site.

India's national TB program covers a population of 1.2 billion through a network of 662 district TB programme units and 2,698 sub-district TB programme units referred to as Tuberculosis units (TUs).Each TU covers a population of approximately 0.5 million. The present study was conducted in 18 selected TUs. Each of the 18 study area TUs has 4–6 designated microscopy centres (DMCs), covering approximately 0.1 million population each, where sputum smear microscopy is available for TB diagnosis. About three to five primary health centers are linked to each microscopy centre, referring presumptive TB cases to the respective microscopy centre. The 18 sites were purposively selected based on the availability of free treatment for patients diagnosed with rifampicin resistance, and to represent diverse geographic and demographic settings across the country. Of these, 8 sites were in rural areas catering to a population of 3.9 million, 6 sites were in urban areas catering to a population of 3.4 million and 4 sites were in tribal and hilly area covering a population of 1.5 million (Fig. 1). Altogether, these 18 sites account for 8.8 million people having access to TB diagnostic services through 99 DMCs and their corresponding linked health facilities.

### Definitions

Presumptive pulmonary TB cases [13] were defined as individuals with symptoms suggestive of pulmonary TB who had sputum samples tested for TB. HIV infected cases with cough of any duration are a suspect for pulmonary TB and are classified as presumptive pulmonary TB cases.

Bacteriologically confirmed pulmonary TB (PTB) [14] cases were defined as patients with a positive test result for TB.

Clinically diagnosed pulmonary TB [14] cases were defined as cases that do not fulfill the criteria for bacteriological confirmation but are diagnosed with active TB by a treating physician—using a standardized programmatic diagnostic algorithm, which incorporates chest X-ray, antibiotic trial, repeat smear microscopy and clinical evaluation of symptoms—and are initiated on TB treatment, as evidenced by registration in a RNTCP (Revised National Tuberculosis Control Program) treatment register.

Pulmonary TB cases were defined as any bacteriologically confirmed or clinically diagnosed case of TB involving the lung parenchyma or the tracheo-bronchial tree. A pulmonary TB case without prior treatment for TB (or less than 1 month of treatment) was considered a new case. Any TB case who had more than one month of anti-TB treatment in the past was defined as previously treated [13].

Presumptive DR-TB case were defined as already diagnosed pulmonary TB cases based on smear-microscopy referred for drug susceptibility testing (DST) because of an elevated risk of drug-resistant TB. National guidelines define high-risk TB cases as those TB cases with previous history of anti TB treatment, TB cases on treatment with positive sputum smear result at any follow up smear examination, diagnosed TB cases with HIV-co-infection and all pulmonary TB cases who are contacts of a known MDR TB case [15].

In this study, at the time of case enrolment, all cases with positive history of TB treatment but who had no confirmed TB diagnosis, either clinically or bacteriologically, are considered as presumptive TB cases.

Rifampicin resistant TB cases were defined as bacteriologically confirmed TB cases with indication of rifampicin resistance on one or more of the following assays: Xpert MTB/RIF, line probe assay (LPA) or phenotypic DST.

### Design

**Fig 2:**
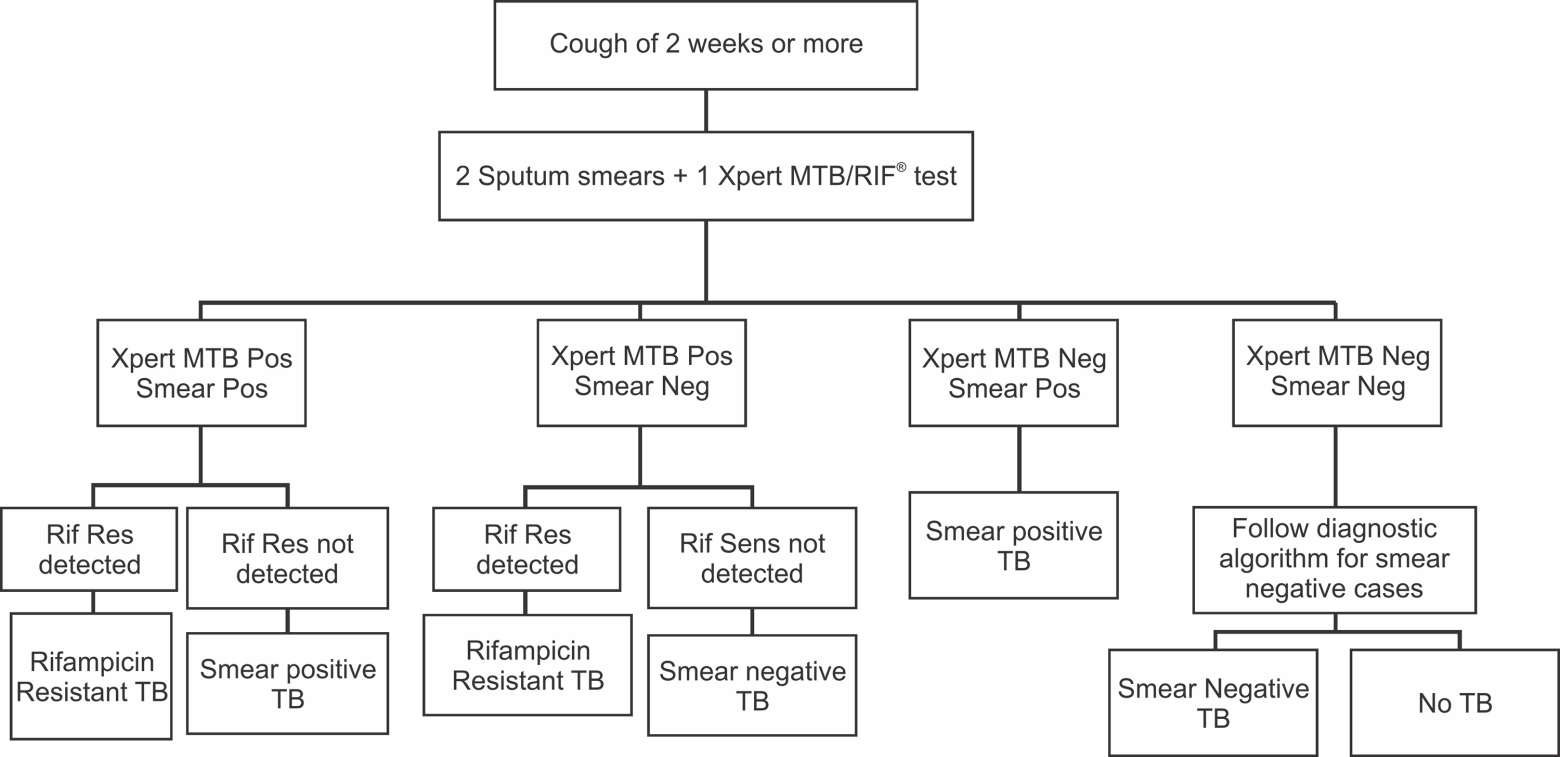
Study diagnostic algorithm.

The current study is a part of a large scale demonstration project, wherein upfront Xpert MTB/RIF testing was offered to all the presumptive TB and presumptive DR-TB cases in the study population. This manuscript focuses on presumptive TB cases known to be HIV infected enrolled in the study. All facilities providing HIV care and support in a given geographical area are linked with DMCs for TB diagnosis and anti TB treatment. Under the study, all these DMCs within TU were linked with respective Xpert MTB/RIF lab. By means of these linkages, all known HIV-infected presumptive TB and presumptive DR-TB cases were provided access to upfront Xpert MTB/RIF testing. Two sputum specimens from HIV-infected presumptive cases were collected at the respective health facility and transported to the Xpert MTB/RIF lab. On receiving samples, initially ZN smear microscopy was performed following which, one of the two samples (1^st^ received sample) was tested on Xpert MTB/RIF using a standardized testing algorithm (Fig. 2)

Treatment of TB and rifampicin resistant TB was initiated based on Xpert MTB/RIF results in line with project diagnostic algorithm as approved by RNTCP. Any case with negative result for TB on Xpert MTB/RIF and a positive smear result on microscopy was managed based on results of smear microscopy. A fresh specimen of these Xpert MTB/RIF negative cases was sent for confirmation of TB diagnosis on solid or liquid culture for analytical purpose. Patients diagnosed as rifampicin-resistant on Xpert MTB/RIF had additional sample collected and sent to identified regional RNTCP DST laboratories for solid/ liquid media DST and line probe assay to confirm Xpert MTB/RIF results. Treatment of these cases was based on initial Xpert MTB/RIF rifampicin resistant result. However any case with rifampicin susceptible results on solid/liquid media DST was subsequently switched to appropriate regimen based on the decision of treating physicians.

To minimize the delay in reporting, Xpert MTB/RIF test results were relayed by Short Messaging Service (SMS) to all health providers and referring facilities. As per the existing RNTCP structure and guidelines, a given TU maintains patient records and undertakes active management and follow-up of diagnosed TB cases that are initiated on treatment at facilities within the same TU area. Under the current study, all consecutive presumptive cases were provided access to project intervention. However, with a small study team, collecting and compiling information on all treatment initiation of diagnosed TB cases that resided outside the study TU was not within the scope of the current study. However, the respective programme officials of the area in which the diagnosed TB cases resided were informed of the details of such cases, as per the RNTCP guidelines and by SMS.

### Data management

The data was collected for all HIV-infected presumptive TB and DR-TB cases using standardized case report forms (CRFs) by the RNTCP staff working at the microscopy centres and TUs. Data from CRFs was entered via a secure, web-based MIS by the site staff. Quality of data was ensured by regular scrutiny of CRFs using cross validation against programme records by project supervisors. Data was extracted and analyzed using EpiData Analysis (Version 2.1) and Microsoft Excel 2007.

### Ethical issues

The study protocol was approved by the Institution Ethics Committee of the National Tuberculosis Institute, Bangalore, India. Structured informed consent forms were used for obtaining written consent from all subjects enrolled in the study. Before taking consent, patients were informed about the study in vernacular language by the trained staff. For illiterate patients, after explaining in their mother tongue, the consent was taken in presence of literate witness. In case of minors/children informed consent was obtained from parents, caretakers, or guardians. Approval for the research was granted by the Central TB Division, Ministry of Health and Family Welfare, Government of India.

## Results

**Fig 3:**
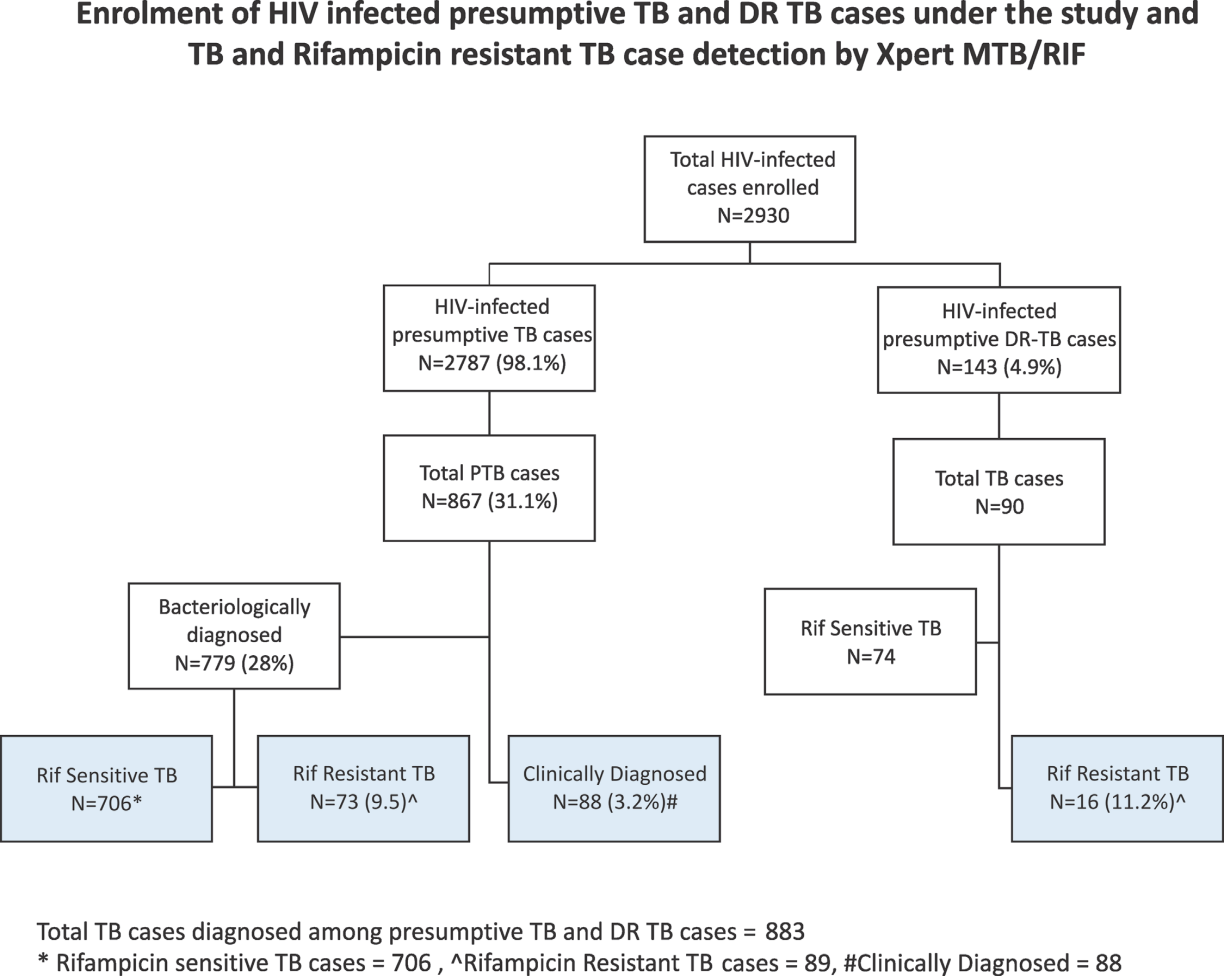
Enrolment of HIV infected presumptive TB and DR TB cases under the study and TB and Rifampicin resistant TB case detection by Xpert MTB/RIF.

A total of 2930 known HIV-infected cases were enrolled across 18 sites from March 2012 to December 2013; 143 (4.9%) presumptive DR-TB cases and 2787 (95.1%) presumptive TB cases. Enrolled presumptive TB cases had male predominance (61.6%); 2689 (96.5%) were in the age group of 15–64 years, about 2/3^rd^ of the enrolled cases were from rural areas and around 4/5^th^ did not have any positive history of prior TB treatment. (Table 1) (Fig. 3).

**Table 1:** Demographic profile of the HIV-infected presumptive TB and DR-TB cases enrolled under the study.

Variables	HIV-infected Presumptive TB case		HIV-infected Presumptive DR-TB case	
	N	%	N	%
Total	2787	100	143	100
Age group (Years)				
0–14	98	3.5	2	1.4
15–24	226	8.1	11	7.7
25–34	814	29.2	43	30.1
35–44	964	34.6	54	37.8
Above 44	685	24.6	33	23.1
Gender				
Female	1071	38.4	35	24.5
Male	1716	61.6	108	75.5
Past history of anti TB treatment				
Not available	1	0	0	0
NO	2206	79.2	7	4.9
Yes	580	20.8	136	95.1
Geographical Area				
Rural	1686	60.5	31	21.7
Urban	885	31.8	111	77.6
Tribal & Hilly	216	7.8	1	0.7

### Xpert MTB/RIF positivity among HIV-infected TB cases

Following the project diagnostic algorithm (Fig. 2), 867 (31.1%, 95% confidence Interval (CI) 29.4‒32.8) HIV-infected TB cases were diagnosed among 2787 presumptive TB cases. 779 (28%, CI 26.3‒29.7) TB cases were bacteriologically confirmed (Table 2), of which 770 (27.6%, CI 26.0‒29.3) TB cases were detected on Xpert MTB/RIF, more than 50% being negative on smear microscopy (Table 3). Remaining, 9 (1.1%) out of the 779 bacteriologically confirmed TB cases were detected on smear microscopy, of which one was not tested and 8 had a negative result for TB on Xpert MTB/RIF. Of these 8 Xpert negative and smear positive cases, additional confirmatory culture results were obtained, of which 3 were found to be culture negative while results for remaining 5 cases was awaited at the time of data analysis. Overall, TB positivity rate on Xpert MTB/RIF observed under the study was 27.6% (CI 26‒29.3) against smear positivity rate of 12.9% (CI 11.7‒14.1). (Table 3) Amongst the bacteriologically confirmed cases, higher detection rates were observed in patients with prior history of TB treatment (36.9% (CI 33.0‒40.8) vs 25.6% (CI 24‒27.4) OR 1.6 (CI 1.3‒2.0)) and patients from urban settings (34.6 (CI 31.5‒37.7) vs 24.8 (CI 22.7‒26.9) OR 1.6 (CI 1.3–1.9)). (Table 2)

**Table 2:** Bacteriologically confirmed TB and all PTB case detection among HIV-infected TB cases.

Variables	HIV-infected presumptive TB cases	Bacteriologically Confirmed TB cases	%	OR (95% CI)	Clinically diagnosed TB cases	%	All PTB cases	%
Total	2787	779	28		88	3.2	867	31.1
Age group (years)								
0–14	98	17	17.3	0.5 (0.3–0.9)	3	3.1	20	20.4
15–24	226	54	23.9	1	6	2.7	60	26.5
25–34	814	229	28.1		19	2.3	248	30.5
35–44	964	267	27.7		34	3.5	301	31.2
Above 44	685	212	30.9		26	3.8	238	34.7
Gender								
Female	1071	223	20.8	1	29	2.7	252	23.5
Male	1716	556	32.4	1.8 (1.5–2.1)	59	3.4	615	35.8
Past history of anti TB treatment								
Not Available	1	0	0			0	0	0
No	2206	565	25.6	1	63	2.9	628	28.5
Yes	580	214	36.9	1.6 (1.3–2.0)	25	4.3	239	41.2
Geographical area								
Rural	1686	418	24.8	1	33	2	451	26.7
Urban	885	306	34.6	1.6 (1.3–1.9)	53	6	359	40.6
Tribal & Hilly	216	55	25.5	1 (0.7–1.4)	2	0.9	57	26.4

*Abbreviations—OR = Odds Ratio*, *CI = Confidence interval*, *PTB = Pulmonary TB*

**Table 3:** Additional gain in detection of bacteriologically confirmed TB cases on Xpert MTB/RIF over smear microscopy.

Variables	Total HIV infected presumptive TB cases	Smear Positivity		Xpert Positivity		Additional gain in detection of bacteriologically confirmed TB cases (Fold increase)
		N	%	N	%	
Total	2787	359	12.9	770	27.6	2.1
Age group (Years)						
0–14	98	8	8.2	17	17.3	2.1
15–24	226	24	10.6	53	23.5	2.2
25–34	814	108	13.3	226	27.8	2.1
35–44	964	128	13.3	264	27.4	2.1
Above 44	685	91	13.3	210	30.7	2.3
Gender						
Female	1071	90	8.4	220	20.5	2.4
Male	1716	269	15.7	550	32.1	2.0
Past history of anti TB treatment						
Not Available	1	0	0	0	0	
No	2206	251	11.4	560	25.4	2.2
Yes	580	108	18.6	210	36.2	1.9
Geographical Area						
Rural	1686	189	11.2	414	24.6	2.2
Urban	885	138	15.6	301	34	2.2
Tribal & Hilly	216	32	14.8	55	25.5	1.7

### Diagnostic turnaround time

Specimen transportation linkages between their respective health facilities and Xpert lab was provided to all presumptive cases. Median diagnostic turnaround time, between specimen collection and availability of test result, was found to be zero day i.e day of specimen collection. It was observed that 2140 (73%) of the cases got test results on the same day of sample collection and another 15% on subsequent day. Cumulatively, 93% of the cases got test result within 2 days and 99% (2911) had received their results within a week.

### Effect of Xpert MTB/RIF upfront testing on rifampicin-resistant TB case detection

Overall 89 rifampicin resistant TB cases were diagnosed; 16 (11.2%, CI 6.7‒17.1) being diagnosed among 143 presumptive DR-TB cases and 73 (9.5%, CI 7.6‒11.8) among the 770 positive TB cases detected on Xpert MTB/RIF. Of the rifampicin resistant TB cases detected among the newly diagnosed TB cases, the detection rates were similar in both smear positive and negative TB cases (9.7%, CI 6.9‒13.1 vs. 9.3%, CI 6.7‒12.3 (OR 1.05, CI 0.6–1.7)), and in both the genders (9.8%, CI 7.5‒12.5 vs 8.6%, CI 5.4‒12.9 (1.1 (CI 0.6–1.9)). (Table 4).

**Table 4:** Rifampicin resistant HIV-infected TB cases diagnosed under the study.

	HIV-infected Presumptive TB cases				HIV-infected Presumptive DR-TB cases		
	Total number of Xpert Positive TB cases	Rifampicin Resistant cases diagnosed on Xpert MTB/RIF	Positivity of Rif resistance on Xpert MTB/RIF (%)	OR (95% CI)	HIV-infected presumptive DR TB cases	Rifampicin Resistant cases diagnosed on Xpert MTB/RIF	Positivity of Rif resistance on Xpert MTB/RIF (%)
Total	770	73	9.5		143	16	11.2
Age group (Years)							
0–14	17	2	11.8	1.2 (0.2–5.7)	2	0	0
15–24	53	4	7.5	1	11	1	9.1
25–34	226	24	10.6		43	7	16.3
35–44	264	23	8.7		54	7	13
Above 44	210	20	9.5		33	1	3
Gender							
Female	220	19	8.6	1	35	5	14.3
Male	550	54	9.8	1.1 (0.6–1.9)	108	11	10.2
Past history of anti TB treatment							
No	560	31	5.5	1	7	1	14.3
Yes	210	42	20	4.2 (2.5–7.0)	136	15	11
Smear Microscopy							
Negative	420	39	9.3	1	83	3	3.6
Positive	350	34	9.7	1.05 (0.6–1.7)	60	13	21.7

*Abbreviations—OR = Odds Ratio*, *CI = Confidence interval*

Of the 89 rifampicin resistant TB cases, confirmatory culture DST/ LPA results were available for 44 patients at the time of analysis and 43 were found to be rifampicin resistant giving a high positive predictive value of 97.7% (CI 89.3‒99.8). The positive predictive value was similar irrespective of history of previous TB treatment (97.1% in patients with past history of anti TB treatment vs 100% among patients with no history of anti TB treatment).

### Treatment initiation and outcome among HIV-infected TB cases

Of a total of 883 TB cases detected under the study (Fig. 3); 89 were diagnosed with resistance to rifampicin, 706 were bacteriologically confirmed TB cases susceptible to rifampicin and 88 clinically diagnosed TB cases. At the time of data closure, positive confirmation of treatment initiation was available for 705 (80%) of the diagnosed TB cases. Of the remaining cases whose treatment information was not available, 111 (13%), were either not the residents of the study area or could not be traced for information of treatment initiation. The median turnaround time for treatment initiation from the date of diagnosis, was found to be 4 days, with 74% of the TB patients being initiated on first line TB treatment within 7days of diagnosis and 73% of rifampicin resistant TB cases being initiated on second line anti TB treatment by 3^rd^ week of diagnosis. We have however excluded 48 patients with no information on exact date of treatment initiation from this analysis of time to treatment. Treatment outcomes for 391TB cases initiated on first line anti-TB treatment were available at the time of study data closure, of which 302 (77.2%) were successfully treated, 23 (5.9%) had defaulted from treatment. A total of 54 (13.8%) deaths and 7 (1.8%) treatment failures were observed (S1 Fig.).

## Discussion

With the introduction of a high sensitivity TB diagnostic tool at the decentralized levels of the health system, more than two fold increases in detection of bacteriologically confirmed TB in comparison to smear microscopy among HIV infected presumptive TB cases was observed. Similar increases have been reported in studies conducted in high HIV prevalent setting of South Africa (Johannesburg) and Ethiopia [16, 17]. This finding further reinforces the usefulness of providing upfront access to Xpert MTB/RIF testing to HIV infected presumptive TB cases, in line with the recently released guidance from WHO [13]. The high sensitivity of Xpert MTB/RIF resulted in detection of significant proportion of smear negative TB cases, with 53% of the total bacteriologically diagnosed TB cases having a negative smear microscopy result. Similar findings were reported from studies conducted in South Africa (Johannesburg) and Ethiopia, where 61% and 68% of the bacteriologically confirmed TB cases, respectively, were smear negative [16, 17].

Factors associated with higher TB detection rates among HIV infected presumptive TB cases were cases with prior history of TB treatment and cases from urban settings. Similar findings have been reported in studies conducted in South Africa (Durban), Nigeria [18, 19]. Given that other studies suggest that patients without bacteriologic confirmation are frequently lost or not initiated on treatment and RIF resistant TB cases remain undetected [20], we believe our findings represents an important advance to ensure early case detection and providing quality TB care.

In view of the challenges in diagnosing TB among HIV infected, offering DST selectively to diagnosed TB cases, can potentially lead to higher morbidity and mortality on account of delays in identification of resistance to rifampicin [20]. In the current study, offering universal upfront Xpert MTB/RIF testing resulted in diagnosis of large number of rifampicin resistant TB cases, with more than four-fifths of the rifampicin resistant cases being diagnosed in presumptive TB cases, more than half of which had a negative smear microscopy result. The finding of the study on high proportion of rifampicin resistance among HIV co-infected smear negative TB patients is reported for the first time from India, supporting the programme decision to extend DST to all PLHIV with PTB, regardless of smear status. High levels of rifampicin resistance among newly diagnosed TB cases, has been reported in studies from Nigeria, Uganda, Peru and South Africa (KwaZulu-Natal) [19, 21, 22, 23]. In smear negative TB cases, other rapid tests for ascertaining rifampicin resistance such as line probe assay have limited application for direct testing on sputum specimen and DRTB diagnosis is largely dependent on Solid or Liquid culture testing, resulting in delayed initiation of appropriate treatment. The benefit of Xpert MTB/RIF in providing quicker diagnosis can thus be extended to provision of timely and appropriate treatment to DR-TB patients which can potentially lead to better treatment outcomes. While the study does not change DST policy for this HIV infected TB cases, it essentially reinforces the policy already adopted by the Government of India, which is to provide DST to all PLHIV with PTB, regardless of smear status. The study demonstrates effective means of implementing this policy for smear negative TB cases.

The high positive predictive value (97.7%) for simultaneous detection of rifampicin was observed under the study is very much similar to the findings from other studies conducted in South Africa (Cape Town), South Korea and Greece [24, 25, 26].

Factors associated with higher level of rifampicin resistant detection were presumptive cases from urban settings (15.4%) and previous history if anti TB treatment (19.1%), no gender specific difference was observed in the rifampicin resistant detection. Our study findings are similar to findings from other studies conducted in Nigeria, and two different studies in South Africa (KwaZulu-Natal and Cape Town) [19, 23, 24].

Operational feasibility of decentralized deployment of Xpert MTB/RIF and its performance from the study has already been documented [27]. A key challenge in providing upfront access to Xpert MTB/RIF testing to PLHIV is the feasibility of establishing effective linkages to cover facilities across a geographic area. Under the study, Xpert MTB/RIF labs were located at decentralized level at a TU health facility and were linked by means of specimen transportation linkages with a large number of health facilities in a given geographic area. The median diagnostic turnaround time (TAT) from the time of collection, was on the same day of specimen collection, with 93% of the cases being provided test results with 2 days of specimen collection. Similar TAT was reported from other studies from Korea and Turkey [28, 29]. However, diagnostic TAT in the current study was lesser than what was observed in another study from South Korea where median TAT was 6 days (3–7 days) in outpatient settings [25]. Taking into account the additional yield both in terms of TB and DR-TB case detection and provision of high proportion of results with a rapid turnaround time, highlights the feasibility and utility of implementation of similar intervention at larger scale. The key reasons leading to test result availability beyond 2 days were specimens being collected on days prior to holidays (Sundays and public holidays) and equipment malfunction.

Tuberculosis remains a leading cause of mortality in HIV-infected patients in sub-Saharan Africa and Asia [30]. Global Tuberculosis report (2013) has reported, 19% death rate among this population group [4]. Various other studies has documented high mortality rates among patient with MDR TB and HIV co-infection [31, 32]. Under the study, significant proportions of diagnosed cases were initiated on treatment and high treatment success rate was documented. The study findings are in-line with findings reported under 2014 RNTCP Annual Status report [33] and other studies from Nigeria and India [34, 35]. Availability of rapid results, with upfront information on rifampicin susceptibility with Xpert testing enable early initiation of appropriate treatment regimen may have contributed to surprisingly good treatment outcomes among PLHIV. Study has reported high treatment success rate, however, with routine ART in policy and widely available and early detection of RIF-resistant TB, still had too-high death rates, which needs to be further investigated.

In line with WHO recommendations on use of Xpert MTB/RIF, this study has captured substantial data on impact of Xpert MTB/RIF as a primary tool for the diagnosis of TB and DR TB cases in HIV-infected groups. Implemented in routine programmatic settings, the findings from the study make a strong case for similar large scale implementation in areas with high prevalence of HIV in TB cases.

Upfront testing on Xpert MTB/RIF offers a distinctive case finding advantage as compared to smear microscopy. However, the high test cost limit and make it challenging to decentralize the availability of Xpert MTB/RIF testing to the level at which smear microscopy is currently available, in the absence of efficient specimen transportation linkages. The current study, demonstrated the feasibility of addressing such implementation challenges in varied field conditions. This crucial aspect should be taken into account while undertaken similar interventions.

These findings and experiences were sufficiently convincing to lead to a change in the national policies, as decided by National TB-HIV working group and standards of TB care, and now all PLHIV are recommended by RNTCP for Xpert for TB detection (where available), and DST for all confirmed TB cases (if not already available from Xpert)

## Conclusion

Our study findings strongly demonstrate limitations of using smear microscopy for TB diagnosis in PLHIV, leading to low TB and DR-TB detection rates in PLHIV and which can potentially lead to delayed or sub-optimal TB treatment initiation. Xpert MTB/RIF offers a promising solution to this diagnostic challenge. Our study demonstrates a striking feasibility of providing upfront Xpert MTB/RIF testing to HIV infected presumptive TB & DR-TB cases, with a rapid turnaround time under field conditions. These findings establish the usefulness and feasibility of efficiently addressing this diagnostic gap with upfront of Xpert MTB/RIF testing, leading to overall all strengthening of care and support package for PLHIV.

## Supporting Information

S1 Fig Treatment outcome among HIV positive TB patients diagnosed under the study. (TIF)

S1 Table Data on all HIV infected TB and DR TB suspects enrolled under the study. (XLSX)
