# Supplementary figures and images for: Catching the Missing Million: Experiences in Enhancing TB & DR-TB Detection by Providing Upfront Xpert MTB/RIF Testing for People Living with HIV in India
Source: PLoS One. 2015 Feb 6;10(2):e0116721. doi: 10.1371/journal.pone.0116721 (PMC4319843; doi:10.1371/journal.pone.0116721)

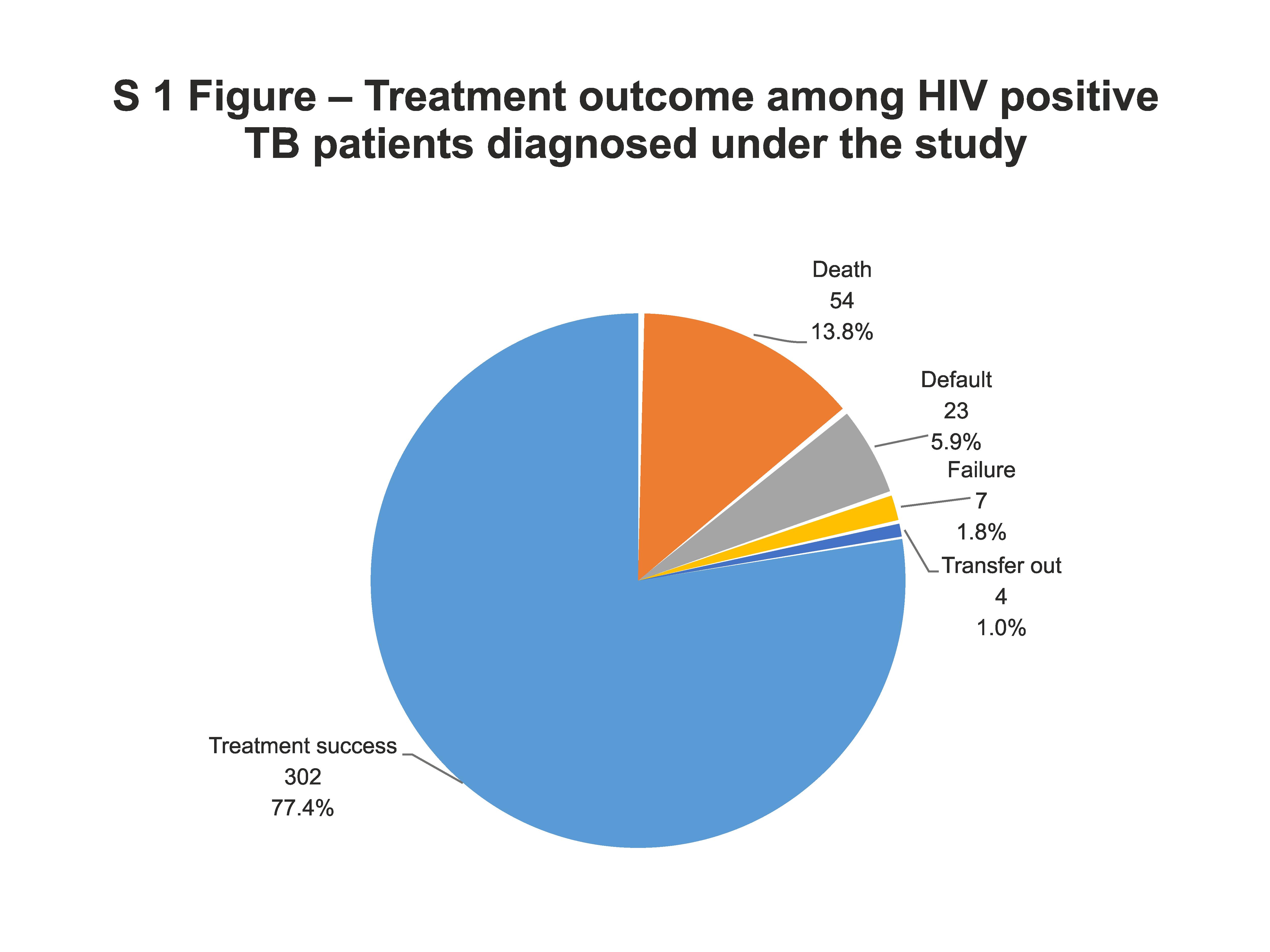

Supplement: S1 Fig — (TIF) [file pone.0116721.s001.tif]
